# Supplementary material for: Neural implementation of computational mechanisms underlying the continuous trade-off between cooperation and competition
Source: Nat Commun. 2022 Nov 11;13:6873. doi: 10.1038/s41467-022-34509-w (PMC9652314; doi:10.1038/s41467-022-34509-w)
Supplement: Supplementary file 1 — Supplementary Information [file 41467_2022_34509_MOESM1_ESM.pdf]

Supplementary Information for

Neural implementation of computational mechanisms underlying the continuous trade-off between cooperation and competition

M. A. Pisauro, E. Fouragnan, D.H. Arabadzhiyska, M.A.J Apps, M. G. Philiastides

*Corresponding author. Email: m.a.pisauro@bham.ac.uk

Supplementary Results

The striatum response in win trials is modulated by the social context.

In our main GLM (GLM 1) we also found significant activation at the time the target was revealed both proportional to the reward available and to weather the trial was won. In particular we found a significant activity in the bilateral striatum (STR; left peak Z=5.43, MNI: x=-14, y=8, z=-12; right peak Z=5.21, MNI: x=16, y=10, z=-12)), Middle Frontal Cortex (MFC; 26 2 56), Middle Cingulate Cortex (MCC; -2 8 28), Anterior Cingulate Cortex (ACC ;-10 34 20), DorsoLateral Prefrontal Cortex (dlPFC; -38 36 14/46 40 14), Orbitofrontal Cortex (OFC; 30 24 -10), bilateral Inferior Temporal Gyrus (IFG; -50 8 18) and Supramarginal Gyrus (SMG; 58 -32 46) at time of reward when averaging together win trials. All regions survived multiple correction fig3a; Z>3.1, whole-brain cluster-based correction P<0.001 (GLM1; Fig3).

The activity pattern represented in this analysis is noteworthy as it shows that the BOLD signal in STR is significantly modulated by the social context (fig.2b; $t_{55} = 3.61$; $p = 0.0006$ for STR). More precisely, we found that the neural activity was higher in the competitive context when participant won more than their partner on a winning trial, compared to the cooperative context where players received an equal split on winning trial (fig3.b) aligning with previous studies [1–3] showing that the presence of potential competitors can shift people's preferences[4–6]. It is important to note, however, that the average reward rates in all three contexts were comparable (supplementary Fig.2d).

Additionally, several areas were significantly modulated by the reward available in each trial. Among them, clusters the Anterior Cingulate Cortex (ACC; Z = 3.68; MNI x = 0, y = 36, z= 16), sgACC (Z = 3.22; -4 34 -10) Mid Cingulate Cortex (MCC; Z = 3.59; -4 -10 34); Posterior Cingulate Cortex (PCC; Z = 3.66; -4 -32 34); ventromedial Prefrontal Cortex (vmPFC; Z = 3.18 -2 26 -26). Interestingly, this latter cluster almost reached significance in the contrast between the cooperative and competitive context (Z = 2.63 2 24 -26). Cortical areas such as the vmPFC and subcortical areas such as the vSTR have been shown to encode value signals in opposite ways in competitive versus cooperative domains [1,7] and their activity is known to be context dependant [8–10]. These mechanisms of contextual modulation have been shown to be linked with activity in the anterior cingulate cortex (ACC) and dorsolateral prefrontal cortex (dlPFC) [11–13]. The ACC in particular, has been shown to allow switching from one strategy to another, more beneficial one [14,15].

A rostro-caudal axis in ACC encodes context dependant changes in cooperation for self and other

We reasoned that the activity of an area which is instrumental to informing the decision-making network about a change in social context ought to be significantly different between the competitive and cooperative context throughout the game. We therefore decided to test for difference in baseline between contexts. Our second GLM model therefore included a single boxcar covering the duration of the trial from its onset to the target appearance. This analysis revealed an area in the dorsal bit of the anterior cingulate cortex which was significantly more active during cooperation than competition (dACC; Z = 3.94, -2 22 28, Supplementary Fig. 5a)

alongside a number of regions which were significantly more active during competition. They were in the Inferior Frontal Gyrus (IFG; Z = - 4.29; 50 20 8), Precentral gyrus (PCG; Z = - 3.5; 48 8 30), and superior parietal lobule (SPL; Z = - 3.4; 42 -56 56).

Interestingly, the dACC cluster was sitting at the inferior end of the dmPFC cluster which was encoding changes of cooperation for self and was modulated by the social context (Fig. 5a). Indeed, even though the activity of this area in dACC when encoding cooperation for self was not significant in the contrast between contexts, analysis of the time-course of its activity revealed that it was active at response time and positively encoded increases in cooperation during the competitive and intermediate context, whereas it was negatively associated with increases in cooperation and therefore positive associated with increases in competition during the cooperative context (Supplementary Fig. 5b). In other words, dACC appears to be signalling “out-of-context” changes of cooperation for self, with its representation shifting with changes in social context.

This area lied posteriorly to the ACCg and PaCg cluster that were signalling the sign of the social prediction error and were also modulated by the social context (Supplementary Fig. 5c). We therefore investigated the hypothesis that context-modulated representation of changes in cooperativeness for self and other was continuously represented along the breadth of ACC with a gradient from self to other along the caudal-rostral axis. Indeed, across all social contexts, the extent to which an increase of cooperation for self is encoded was declining moving anteriorly along the ACC. Conversely, the representation of increases of cooperation for the other player was increasing along the same axis. Both effect were more pronounced in the competitive context compared to the cooperative one (Supplementary Fig. 5d).

We reasoned that if the ACC represents context-modulated changes in cooperativeness with a self-other gradient along the rostro-caudal axis, this should be reflected by the parameters of our model. We therefore correlated the parameter representing the *social bias*, capturing the degree to which participants’ behaviour was biased towards cooperation, with the average *betas* of the three ACC clusters for the player increases in cooperation at time of response. We performed the same analysis with the sign of the social prediction error at the time the co-player response is revealed. Similarly, we reasoned that the same would apply for the *titXtat* parameter, capturing the degree to which participants’ behaviour was determined by the attempt to reciprocate the level of cooperation of the co-plater. Corroborating our previous findings we found that the representation of increases of cooperation for self positively correlated with the *social bias* parameter for all three ACC clusters (Supplementary Fig. 5e, left column). Conversely, we found that the strength of the representation of increases of cooperation for self negatively correlated with *titXtat* parameter for all three ACC clusters and increasingly so while moving forward along the ACC (Supplementary Fig. 5e, right column). While we did not find any significant correlation between the model parameters and social prediction error encoding, these findings provide further evidence that a rostro-caudal axis exists whereby more posterior part of the ACC encodes self-regarding cooperation as a fixed policy while more anterior areas take into account the co-player choices and their reciprocation.

Finally, to further investigate how the social context modulated these correlations, we looked at how betas for the three ACC clusters capturing the strength of the representation of increases of cooperation for self, correlated with the *social bias* and *titXtat* parameters in the different contexts. Interestingly, we found that the correlation with the *social bias* decreases in the

cooperative and intermediate context as we progress towards the most anterior bits of ACC whilst in the competitive context it increases (Supplementary Fig. 5f, left column). Conversely the anticorrelation with titXtat also decreases in the cooperative and intermediate context while progressing towards the most anterior bits of ACC whilst in the competitive context it increases (Supplementary Fig. 5f, right column). Contrary to the correlation of the betas averaged across contexts, the only correlations which are significant for the *social bias* are for the competitive condition for ACCg and PaCg. Whilst the only correlations which are significant for the *titXtat* parameter are in the competitive context for PaCg and in the intermediate context for ACCg (Supplementary Fig. 5f). However, this analysis provides further evidence that the social context changes how ACC encodes changes of cooperation for *self*.

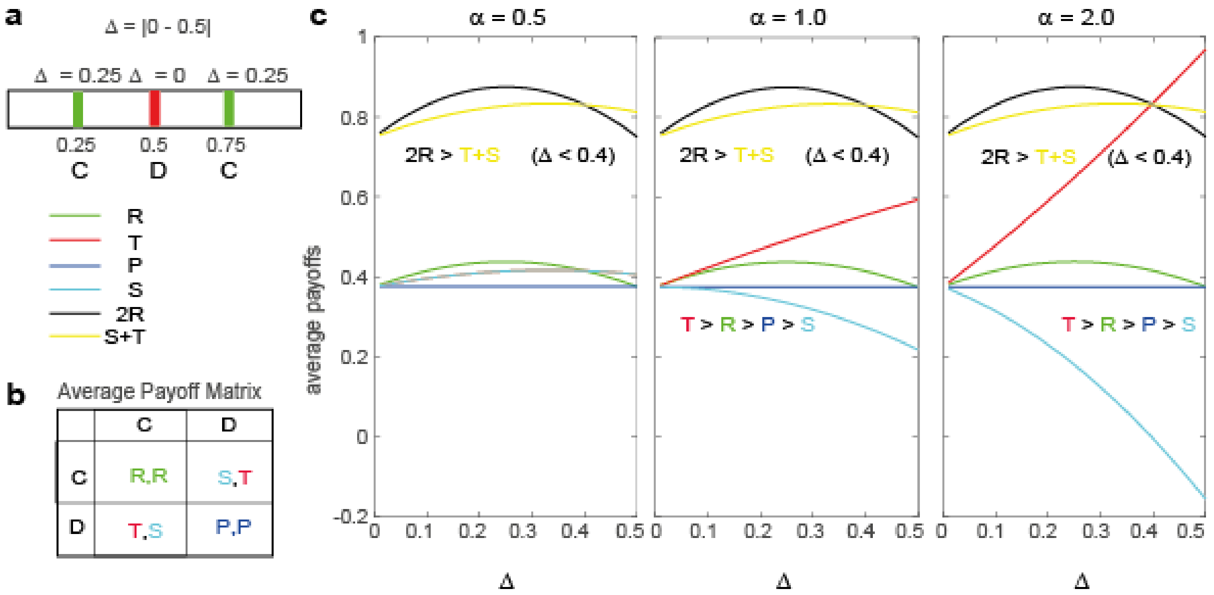

Supplementary Fig.1. Schematic representation of the payoff matrix. (a) We define Δ as the distance from the midpoint and the optimal cooperation **C** as occupying the centre of either hemifield. The optimal way to compete to win the trial and defect cooperation (**D**) is to occupy the midpoint. **(b)** We can define an average payoff matrix with the average reward obtained by players that stick to these two stereotypical orientation over numerous trials while the target position is randomly chosen from a uniform distribution over the space. *R* is the average reward when two players always cooperate. *T* is the "temptation" payoff for someone who goes for the optimal competition position against optimal cooperation. *S* is the "sucker" payoff for a cooperator betrayed by the partner. *P* is the "punishment" payoff when both players compete all the time. (**c**) In all conditions, for almost all positions in the space ($\Delta < 0.4$), the payoff for a dyad always cooperating (2R) is always higher than for one where one player is always competing and other always cooperating or if both alternate cooperation and competition (2*R* > *T* + *S*). Furthermore, for all conditions the maximum payoff for the dyad is reached for $\Delta = 0.25$. For the intermediate and competitive context, for all values of Δ it is also true that competing against a cooperator is more rewarding than cooperating (*T*>*R*), and that cooperating with a competitor is always worse than competing (*S*<*P*), but cooperating with a cooperator is better than competing against a competitor (*T* > *R* > *P* > *S*). Notice that in the competitive condition, the temptation *T* is significantly higher than *R* for all values of Δ and increasingly so for higher values of Δ. The loss associated with cooperating in competitive context (the difference between *R* and *S*) also increase with Δ discouraging cooperation in this condition. In the cooperative condition, the *T* and *S* curves overlap as the reward is always shared. As a result, competing in this condition is rewarding only if the co-player takes extreme positions (for $\Delta > 0.4$).

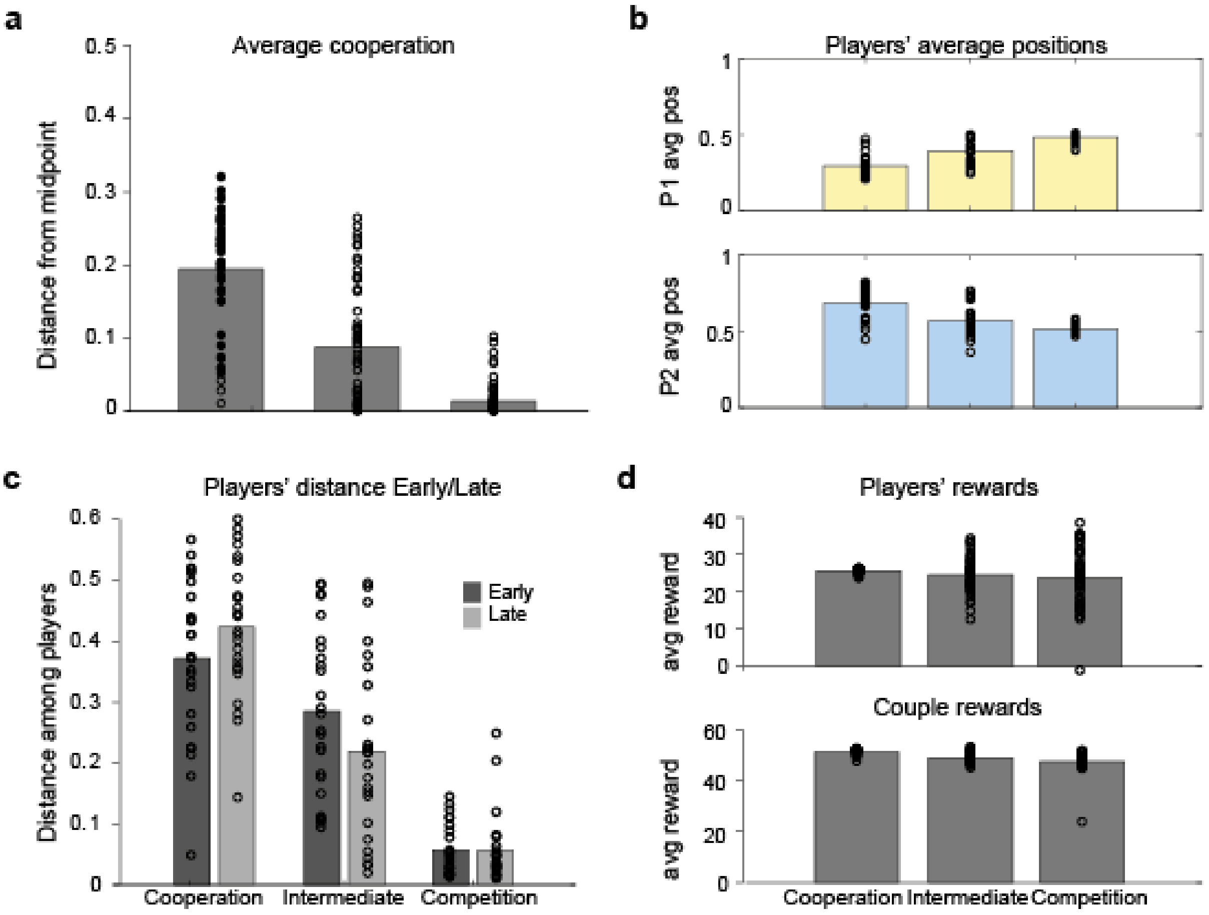

Supplementary Fig2. Behavioural responses. (a) Behaviours in the task depend on the social context and on individual biases. In the cooperative condition, players on average positioned themselves close to the middle of the hemifields (around 0.2 away from the midpoint in either hemifield). In the intermediate condition, players on average switched between cooperation and competition, resulting in a position closer to the midpoint (around 0.1). In the competitive condition, players on average aimed for the middle of the territory. In all conditions N = 50. **(b)** Average position split by players occupying different hemifields. The distance between the players decreases from cooperation to competition. In all conditions N = 25. **(c)** In the cooperative condition, participants slightly increase their distance in later trials, aiming at perfect cooperation. In the intermediate condition, they became more competitive in the later part of the block whereas in the competitive condition, their behaviours remained stable across the trials. In all conditions N = 25. **(d)** Average reward rates in three contexts for players (N = 50) or couples (N = 25). Please notice that one couple had significantly lower rewards in competition due to the scan being interrupted after 30 trials for a technical glitch.

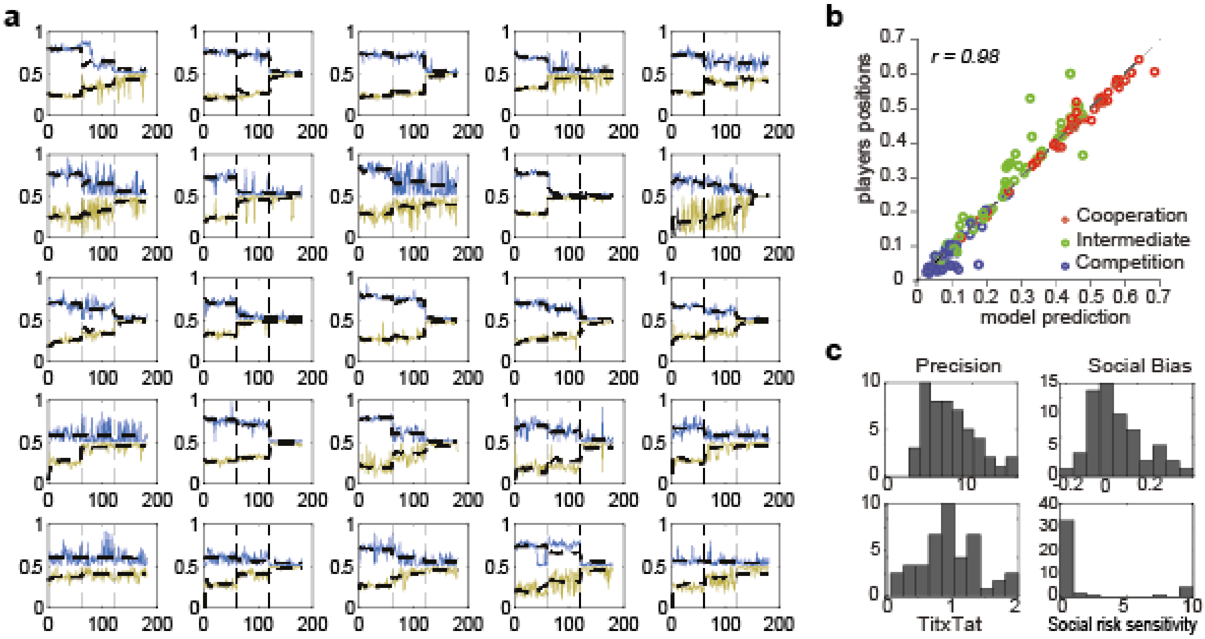

Supplementary Fig.3. Behavioural responses and model fit. (a) All pairs of players are represented in blue and yellow. The model fit is superimposed in black. **(b)** Scatter plot showing linear correlation between the empirical data and predicted average choice positions for each participant and context **(c)** Distribution of all parameters of the full model fitted to behaviours.

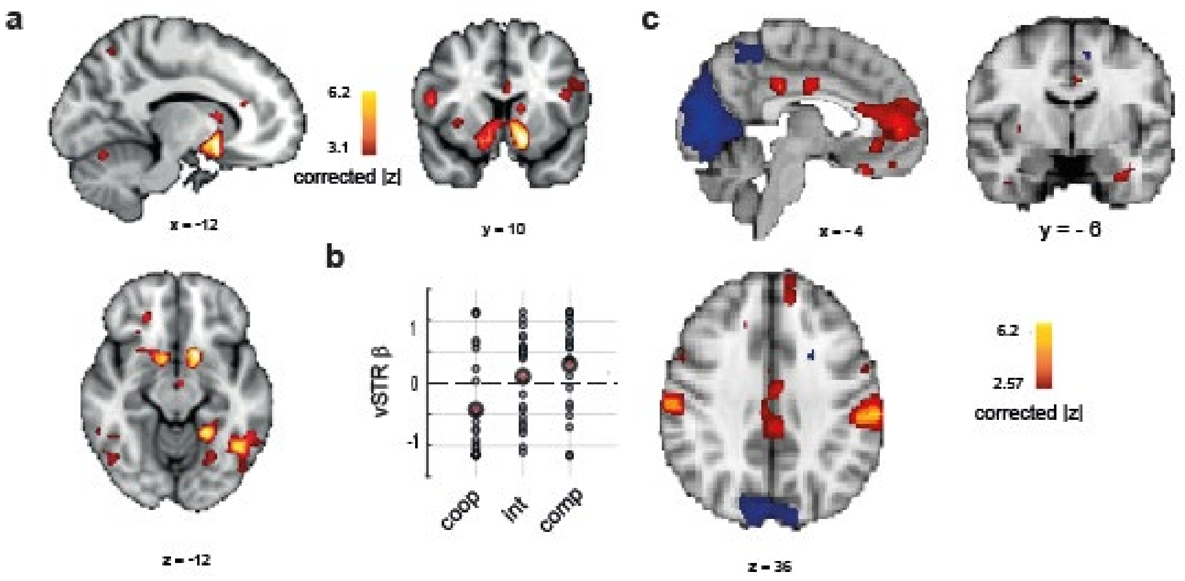

Supplementary Fig4. The striatum showed different response to reward and other's strategy depending on the social contexts. (a) A whole-brain analysis tested for voxels which were active in trials won by the player inside the scanner, at the time the winner was revealed. The fMRI analysis was time-locked to the reward delivery (cluster-corrected, $|Z| > 3.1$, two sided $P < 0.05$). **(b)** The average signal of the striatal activation for all three social contexts showed a significant effect of context, with a higher striatal response for the competitive context compared to the cooperative context. **(c)** A whole-brain analysis tested for voxels which were active at the time when the target's position was revealed and were modulated by the amount of reward available.

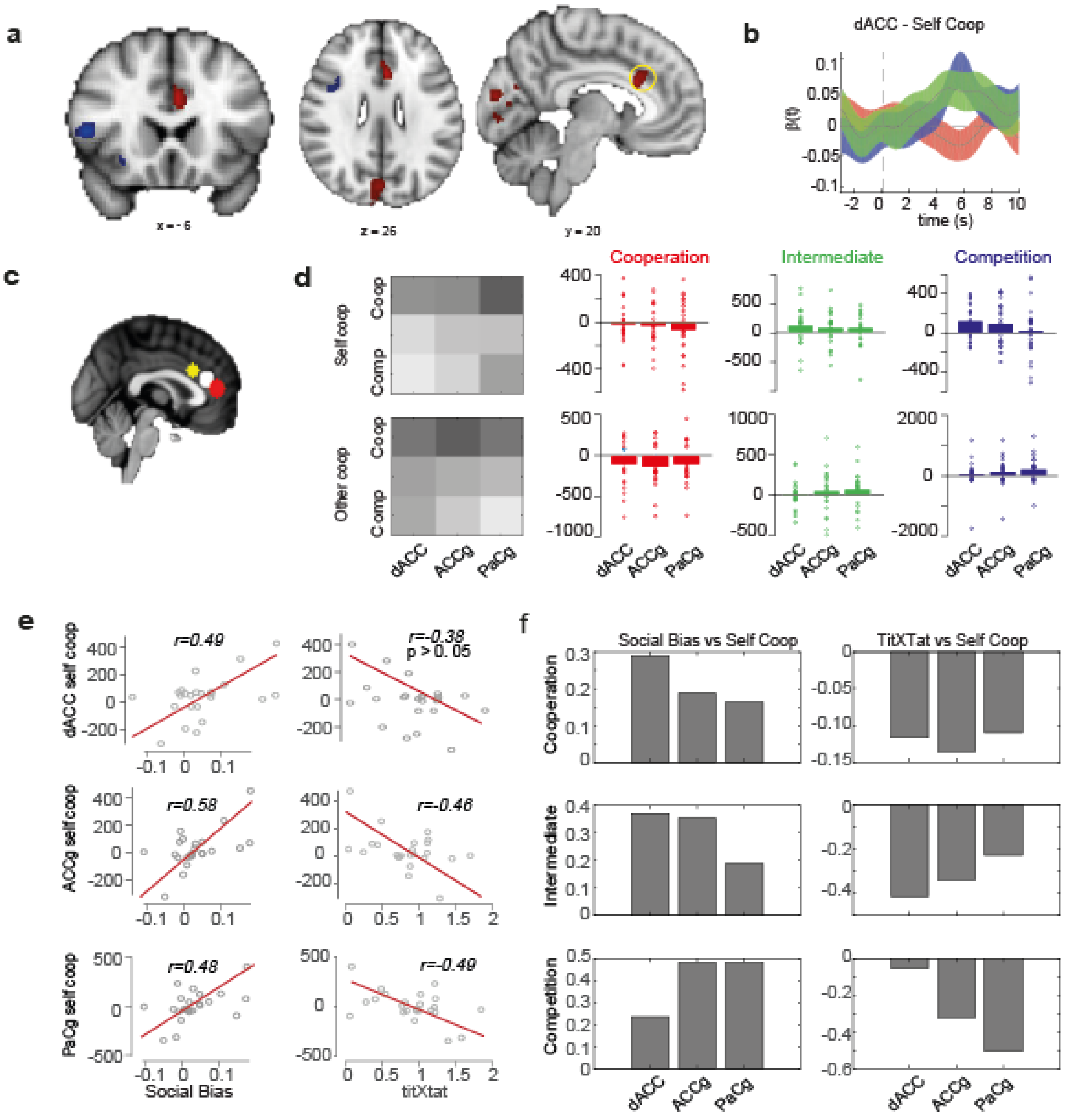

Supplementary Fig.5. A rostro-caudal axis in ACC encodes context dependent changes in cooperation for self and other (a) A whole-brain analysis tested for voxels which were had significantly different activations between the cooperative and competitive context, throughout the trial. The fMRI analysis was time-locked to the start of the trial and looked at activity till the appearance of the target (cluster-corrected, $|Z| > 3.1$, two sided $P < 0.05$). **(b)** The degree to which dACC encoded increases in players' cooperation differed according to the social context. Increases in cooperation were positively associated with activity in dACC in the competitive and intermediate context, whereas they were negatively associated with dACC activity during cooperation. Traces are population averages (+/- SEM). **(c)** ROIs for the three clusters in dACC,

ACCg and PaCg. **(d)** Strength of the average representation of increases in self (top row) and other coop (bottom row), divided by context and cluster ROI. The 3x3 matrices on the left show the average betas for the three ACC clusters capturing the strength of the representation of increases of cooperation for self and the signed prediction error for the co-player's increase in cooperativeness. In the plots on the right, each dot represents the beta from an individual participant, while the bar represents the population average as in the matrices (N= 25). **(e)** Scatter plots showing significant correlations between model parameters and average betas for changes in cooperation for *self* for the three ROIs. Each dot represents a participant (N = 25). The betas are averaged across contexts. All correlations are significant (one sided $P < 0.05$ for a correlation higher than r) but that between dACC and TitXtat. **(f)** Correlation coefficients between the strength of the representation of increases of cooperation for self and the *social bias* (left column) or *titXtat* parameter (right column) for the three clusters and contexts.

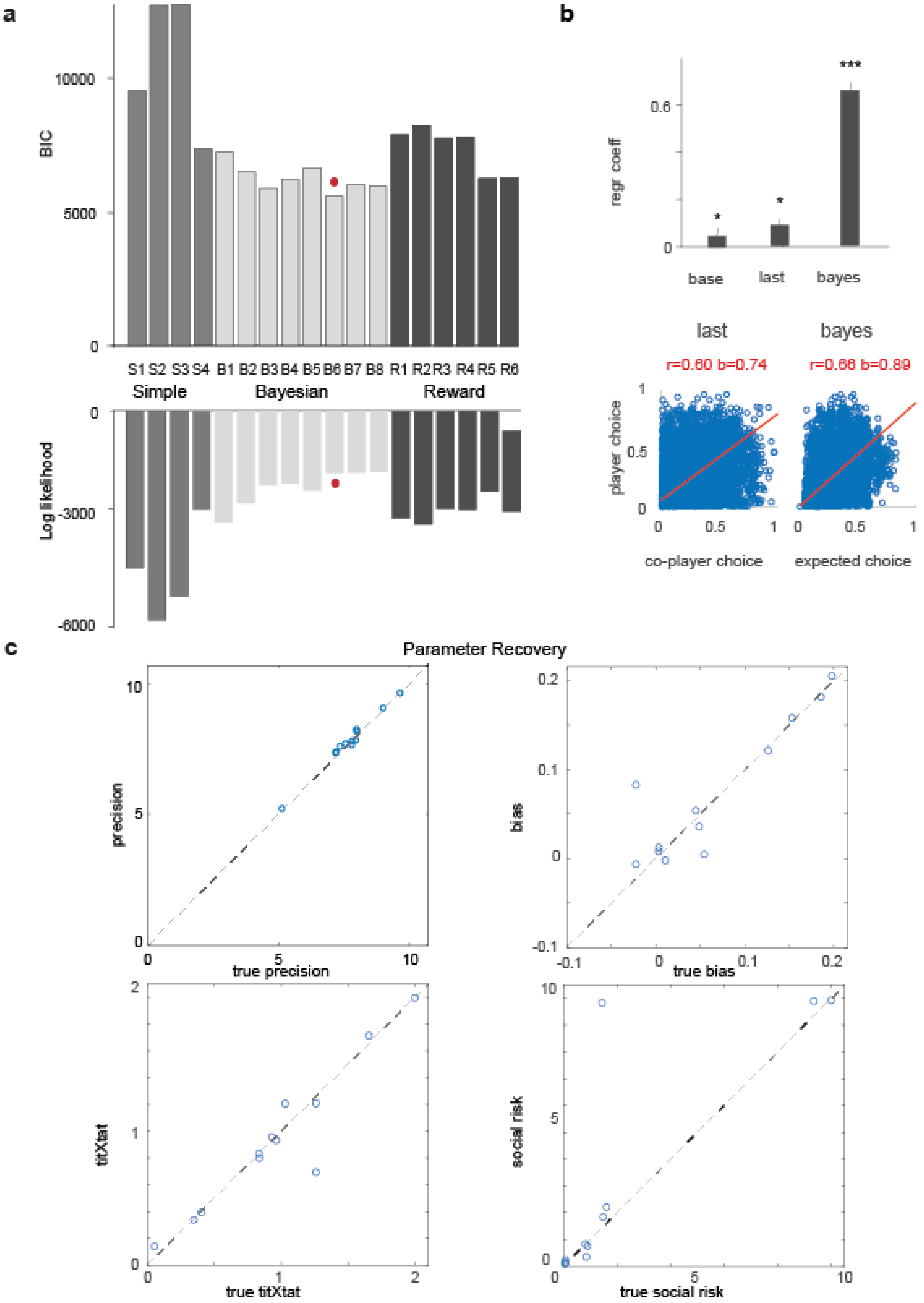
a
BIC
10000
5000
0
S1 S2 S3 S4 B1 B2 B3 B4 B5 B6 B7 B8 R1 R2 R3 R4 R5 R6
Simple
Bayesian
Reward
Log likelihood
0
-3000
-6000
b
regr coeff
0.6
0
*
*
***
base
last
bayes
last
bayes
r=0.60 b=0.74
r=0.66 b=0.89
player choice
1
0.5
0
0
0.5
1
co-player choice
expected choice
c
Parameter Recovery
precision
true precision
bias
true bias
titXtat
true titXtat
social risk
true social risk

Supplementary Fig.6. Model selection, validation and parameter recovery. (a) Bayesian Information Criteria (BIC) and Log Likelihood computed for all models. Models are divided into three classes, 'Simple' (S1-4), 'Bayesian' (B1-8) and 'Reward' (R1-6) based on their underlying logic (see text). The Bayesian model B6 (indicated by a red dot) with context-modulated tit-for-tat and social bias performs best, across the three separate blocks (*BIC*=5553; *LL* = -1866). **(b)** Regression coefficient (+/- 95% confidence intervals) of the players' choices based on the corresponding trial expectation of the co-player position ('bayes') or their last position ('last') according to the model: choice ~ b_0+ b_1*last + b_2*bayes. Whilst both are significant predictors, the expectation predicts the choice better than the observation (suggesting a prediction explain choices better than a reaction). This result is corroborated by comparing the correlation coefficient of the player's choice with the co-player last choice or their expected choice (bottom plots). All analysis are based on correlating all choices from all players in all sessions (N = 59 trials x 3 conditions x 50 participants = 8850; the first trial is excluded from this analysis as players choose without knowledge of co-player strategy). Significance is computed through one-sided *t*-tests for regression coefficient being significantly different from zero. *Significance at P <0.05 *** P<0.001. **(c)** Results from a parameter recovery analysis. Average precision, bias, titXtat and social risk parameters respectively, recovered for six different simulated data sets. Each dot is the average of 10 fits of the simulated data set. Most parameters were recovered reliably – note similarity between simulated (*y*-axis) and true parameters (*x*-axis).

SUPPLEMENTARY TABLES

Supplementary Table 1

	S1	S2	S3	S4	B1	B2	B3	B4	B5	B6	B7	B8	R1	R2	R3	R4	R5	R6
BIC	9484	12647	12683	7295	7184	6463	5845	6169	6606	**5553**	5995	5933	7841	8181	7709	7749	6213	6241
-LL	4733	6305	5584	2965	3364	2776	2239	2173	2392	1866	1859	1828	3238	3408	2944	2964	2424	2438

BIC values and Negative Log Likelihood for all models.

Supplementary Table 2

Region	Hemisphere	Peak MNI coordinates (mm) X	Y	Z	Z Value (Peak)
absPE clusters (average)					
Posterior Temporal Parietal Junction	R	52	-58	30	4.40
Inferior Frontal Gyrus	R	50	16	14	4.06
Middle Temporal Gyrus	R	56	-30	-10	3.90
Middle Temporal Gyrus	R	60	4	-24	3.73
Middle Frontal Gyrus	R	44	16	40	3.65
Insula	L/R	±34	22	-4	3.40/3.60
Lateral Occipital Cortex	L	44	-68	46	3.20
Precentral Gyrus	L/R	±18	-22	70	-4.32/-4.47
Parietal Operculum Cortex	R	48	-28	24	-3.62
Occipital Pole	L/R	±22	-96	-4	-3.76/-3.86
absPE clusters (contrast)					
-					
signPE clusters (average)					
Anterior Temporal Parietal Junction	R	50	-38	32	-3.67
Lingual Gyrus	L	-12	-82	-10	3.41
signPE clusters (contrast)					
ParaCingulate Gyrus	R	2	50	12	-3.36
Anterior Cingulate Gyrus	R	4	32	20	-3.13
PriorPos clusters (average)					
Frontal Pole	L	-24	42	14	3.91
Frontal Pole	R	30	46	14	3.43
PriorPos clusters (contrast)					
Lateral Occipital Cortex	L	-34	-86	0	3.50
Pcoop clusters (average)					
Occipital Pole	L	0	-92	2	4.06
Lingual Gyrus	R	22	-62	2	4.34
Pcoop clusters (contrast)					
Dorsomedial Prefrontal Cortex	L	-8	16	52	-4.09
Precuneous Cortex	L	-6	-56	56	-3.95

Insula	R	30	26	0	-3.85
Superior Frontal Gyrus	R	28	6	56	-3.54
Rew clusters (average)					
Anterior Cingulate/Paracingulate Gyrus	L	-2	52	4	3.79
Posterior Cingulate Cortex	L	-4	-32	34	3.65
Mid Cingulate Gyrus	L	-4	-10	34	3.59
Subgenual Anterior Cingulate Cortex	L	-4	34	-10	3.22
VentroMedial Prefrontal Cortex	L	-2	26	-26	3.18
Occipital Pole	R	34	-90	-2	5.87
Occipital Pole	L	-30	-94	12	5.03
Lingual Gyrus	L/R	±12	-82	-10	-5.67/-5.22
Rew clusters (contrast)					
Superior Parietal Lobule	R	38	-44	46	-3.49
Inferior Frontal Gyrus	R	38	28	12	-3.42
WIN clusters (average)					
Putamen	L	-14	8	-10	5.66
Putamen	R	16	10	-12	5.21
Supramarginal Gyrus	L/R	±58	-32	46	5.62/4.23
Inferior Frontal Gyrus	R	±50	8	18	4.69/4.80
Orbital Frontal Cortex	R	30	24	-10	4.42
DorsoLateral Prefrontal Cortex	L	-38	36	14	4.28
DorsoLateral Prefrontal Cortex	R	46	40	14	3.72
Middle Cingulate Cortex	L	-2	8	28	3.66
Anterior Cingulate Cortex	L	-10	34	20	3.46
Middle Frontal Cortex	L	-26	2	56	4.17
Precentral Gyrus	L	-52	6	38	5.56
Lateral Occipital Cortex	L	-46	-66	-4	4.84
WIN clusters (contrast)					
Middle Frontal Gyrus	R	48	28	26	-3.34
TRIAL clusters (average)					
Frontal Pole	R	34	56	-12	4.12
Frontal Pole	L	-48	38	-12	4.23
Frontal Orbital Cortex	L/R	±10	30	-26	3.89/3.57
Lateral Occipital Cortex	L/R	±52	-60	46	5.18/4.75
Precentral Gyrus	L/R	±26	10	46	4.27/4.17
Supramarginal Gyrus	L/R	±66	-24	24	-3.71/-4.42
Cingulate Gyrus	R	4	32	22	-5.08
Postcentral Gyrus	L.R	±22	-40	64	-5.01/-5.50
Inferior Temporal Gyrus	L/R	-42	-40	-20	-5.93
Occipital Pole		0	-88	12	-6.76
PR clusters (average)					
Precentral Gyrus	L/R	±46	-6	58	5.53/3.48
Postcentral Gyrus	R	62	-18	36	5.51
Lobule Cortex		0	0	54	5.36
Putamen	L/R	±26	8	-2	5.76/4.21
Inferior Frontal Gyrus	R	54	12	16	5.15
Supramarginal Gyrus	R	44	-40	48	6.01
Occipital Pole		0	-94	24	-4.94

OR clusters (average)					
Superior Frontal Gyrus	R	4	38	42	4.10
Lobule Cortex		-2	2	54	-5.74
Frontal Pole	L/R	-36	52	32	-4.16
Insula	L/R	±34	4	8	-4.09
Postcentral Gyrus	L/R	±24	-36	62	-3.68/-3.87
Superior Temporal Gyrus	L/R	±52	-8	-10	-3.69/-5.45
Frontal Pole	L	-26	38	-10	-4.1
TARGET clusters (average)					
Insula	L/R	±38	18	-6	4.94/5.35
Frontal Gyrus	R	38	52	8	4.71
Lateral Occipital Cortex	L/R	±48	-78	-8	5.26/7.02
Supramarginal Gyrus	L/R	±48	-40	44	5.34/6.53
Putamen	L	-24	8	2	4.30

Supplementary Table 2. BOLD activations in the 10 predictors of our GLM I. Complete list of activations correlating negatively or positively with the single-trial variability in our *absPE* (absolute value of the social prediction error), *signPE* (sign of the social prediction error), *PriorPos* (expected position of the co-player based on the prior), *Pcoop* (Player chosen level of cooperation), *Rew* (total trial reward) parametric regressors, *WIN* (whether participant won +1 or lost -1), and the four unmodulated regressors *TRIAL* (at trial start), *PR* (at player response), *OR* (at opponent response), *TARGET* (at target appearance). We also report the areas significantly different in the contrast between the cooperation and competition conditions for our parametric regressors. (GLM; mixed effects, $|Z| > 3.01$, corrected). MNI, Montreal Neurological Institute; L, left hemisphere; R, right hemisphere. Coordinates at peak, for bilateral clusters, coordinates of the highest peak.

Supplementary Table 3

		Peak MNI coordinates (mm)			
**Region**	**Hemisphere**	***X***	***Y***	***Z***	**Z Value (Peak)**
Boxcar clusters (average)					
Insula	L/R	±30	22	-8	3.83/4.51
Putamen	L/R	±18	0	-8	4.22
Frontal pole	L/R	0	56	-18	3.94
Brain stem	R	0	-16	-16	3.89
Inferior Temporal Gyrus	L	-60	-30	-20	3.55
Supramarginal Gyrus	L/R	38	-40	42	5.30

Occipital Pole	L/R	0	-96	2	-6.51
Temporal Pole	L/R	26	6	-22	-3.64
Boxcar clusters (contrast)					
Anterior Cingulate Cortex	L	-4	22	28	3.96
Supracalcarine Cortex		0	-84	8	3.50
Precentral Gyrus	R	48	8	30	-3.40
Superior Parietal Lobule	R	42	-56	56	-3.51
Inferior Frontal Gyrus	R	50	20	8	-4.29

Supplementary Table 3. BOLD activations in the predictor of our GLM II. Complete list of activations correlating negatively or positively with the single-trial variability in our boxcar regressor covering the duration of the trial. (GLM; mixed effects, $|Z| > 3.01$, corrected). MNI, Montreal Neurological Institute; L, left hemisphere; R, right hemisphere.

Supplementary Table 4

**Did you follow a particular strategy in condition A? *Please add details***	**Did you follow a particular strategy in condition B? *Please add details***	**Did you follow a particular strategy in condition C? *Please add details***
I tried to stay on one side of the midpoint and each turn went closest to where my opponent last was.	I tried to stop closest to where my opponent last was but on the opposite side and stayed near the midpoint.	Eventually I tried going between the last winner and the food each time but I couldn't really work out a strategy.
Take 1/3 of position	Tried to to take 1/3 of position, other player did not respond so positioned in the middle.	Positioned in the middle, tried to see if other player would respond to change in positions at 1/3.
Sticking to the left side	Varying position but staying near middle	Varying position but often going to the middle
Yes, cooperative	Yes, gave the other participant 5 trials to cooperate and when he did not, competitive	Yes, competitive
Yes, aiming for opposite ends.	Yes, competing for the middle.	No, it was completely random.
Yes, I tried to stick too one hemisphere in order to maximze the possibilities of a higher score for my partner and me.	Yes, I tried to stick to the midline to one quarter of the right hemisphere as well as to predict the possible moves of my partner based on their previous ones. Therefore, I changed my positions slightly sometimes as I wanted to give myself an advantage.	Yes, I tried to stick to the midline in order to win "one side", to give myself a tiny bit of an advantage to my partner

to stay in the other participants half	to stay around the middle, but adapted to the other participant by trying to stay around her	to stay around the middle, but adapted to the other participant by trying to stay around her
Yes, keeping on the middle of the own half	Yes, going up to 75% of own half towards the middle	Yes, try to go to the middle
After figuring out which player would guess on which side, both myself and the other player guessed a third or two thirds of the way into the scale, maximizing reward for both players.	I alternated guessing the midpoint and guessing slightly further into each half of the scale to throw off my competitor.	I tried to guess as close to the midpoint as possible
Yes, placing myself in the middle of the left hemifield.	Yes, same as in A	Yes, placing myself in the middle of the field.
I placed myself 25% of the way across the field in order to maximize the reward we would get collectively.	I placed myself 25% of the way across the field in order to maximize on the reward within my own half of the field.	I placed myself in the middle in order to minimize the number of losses incurred by my opponent winning
Go 3/4 down the line	Fairly close to the middle but okay to be a bit further away than condition C	As close to the middle as possible
I played in just of the two hemifield, paying attention to the other player's moves	I played at times trying to go for the mid point, others for one of the two hemifields.	I tried to go for the mid point to try to get the max reward
Yes, we spread the space with my co-player.	I tried to be in the middle, as precisely as possible.	We spread the space again, I tried to be in the middle of my area.
75% of the way across the line (midpoint of right hemiside)	aimed to maximise my chances by going close to opponent creating most distance for myself	midpoint
Most often go towards the centre of either hemifield	Most times go towards the centre of either hemifield to maximise the chances of winning	Aim towards the centre of the space to minimise the distance from the target
remained at 2 different ends of the bar	tried to co-operate with the partner, however it did not always work, so then aimed to stay in the middle	aim for the midpoint
Yes. Randomly changed positions from left to centre to right	Same as condition A	No
I tired to keep to one side while the other player kept to the other one	I tried to stick to the middle and the other player seemed to do so as well	I tired to keep to one side while the other player kept to the other one
Divide the field with the other player	At first I tried to divide the field and then just tried to go at either side close to the middleGo for the middle to maximize the chance of success	Go for the middle to maximize the chance of success
I tried to be at 75% of the bar. However, for the later blocks of A I was in the middle. This was not	I stayed roughly at around 75%	I stayed at the middle (50%)

a strategy but because I mistakenly thought it had moved on to condition C.		
Yes. I tried to stop the line in the middle.	Yes. I stopped the line in the middle.	No. I randomly stopped the line based on the previous position.

Supplementary Table 4. Participants' response to the post-experiment questionnaire.

Supplementary references

1. Fliessbach, K. *et al.* Social comparison affects reward-related brain activity in the human ventral striatum. *Science (80-.).* **318**, 1305–8 (2007).
2. Bault, N., Joffily, M., Rustichini, A. & Coricelli, G. Medial prefrontal cortex and striatum mediate the influence of social comparison on the decision process. *Proc. Natl. Acad. Sci. U. S. A.* **108**, 16044–16049 (2011).
3. Fareri, D. S., Niznikiewicz, M. A., Lee, V. K. & Delgado, M. R. Social network modulation of reward-related signals. *J. Neurosci.* **32**, 9045–9052 (2012).
4. Wilson, M. & Daly, M. Do pretty women inspire men to discount the future? *Proc. R. Soc. B Biol. Sci.* **271**, S177-9 (2004).
5. Bault, N., Coricelli, G. & Rustichini, A. Interdependent utilities: How social ranking affects choice behavior. *PLoS One* **3**, e3477 (2008).
6. Ermer, E., Cosmides, L. & Tooby, J. Relative status regulates risky decision making about resources in men: evidence for the co-evolution of motivation and cognition. *Evol. Hum. Behav.* **29**, 106–118 (2008).
7. Fehr, E. & Krajbich, I. Social Preferences and the Brain. in *Neuroeconomics: Decision Making and the Brain: Second Edition* 193–218 (2013). doi:10.1016/B978-0-12-416008-8.00011-5
8. Rangel, A. & Clithero, J. A. Value normalization in decision making: Theory and evidence. *Current Opinion in Neurobiology* **22**, 970–981 (2012).
9. Seymour, B. & McClure, S. M. Anchors, scales and the relative coding of value in the brain. *Current Opinion in Neurobiology* **18**, 173–178 (2008).
10. Philiastides, M. G., Biele, G. & Heekeren, H. R. A mechanistic account of value computation in the human brain. *Proc. Natl. Acad. Sci. U. S. A.* **107**, 9430–5 (2010).
11. Sanfey, A. G., Rilling, J. K., Aronson, J. A., Nystrom, L. E. & Cohen, J. D. The neural basis of economic decision-making in the Ultimatum Game. *Science (80-.).* **300**, 1755–1758 (2003).
12. Tabibnia, G., Satpute, A. B. & Lieberman, M. D. The sunny side of fairness: Preference for fairness activates reward circuitry (and disregarding unfairness activates self-control circuitry): Research article. *Psychol. Sci.* **19**, 339–347 (2008).
13. Apps, M. A. J., Rushworth, M. F. S. & Chang, S. W. C. The Anterior Cingulate Gyrus and Social Cognition: Tracking the Motivation of Others. *Neuron* **90**, 692–707 (2016).
14. Kolling, N., Behrens, T. E. J., Mars, R. B. & Rushworth, M. F. S. Neural mechanisms of foraging. *Science (80-.).* **335**, 95–98 (2012).
15. Fouragnan, E. F. *et al.* The macaque anterior cingulate cortex translates counterfactual choice value into actual behavioral change. *Nat. Neurosci.* **22**, 797–808 (2019).
