# Supplementary material for: Neural implementation of computational mechanisms underlying the continuous trade-off between cooperation and competition
Source: Nat Commun. 2022 Nov 11;13:6873. doi: 10.1038/s41467-022-34509-w (PMC9652314; doi:10.1038/s41467-022-34509-w)
Supplement: Supplementary file 3 — Reporting Summary [file 41467_2022_34509_MOESM3_ESM.pdf]

Corresponding author(s): Michele Andrea Pisauro

Last updated by author(s): 27/6/22

Reporting Summary

Nature Portfolio wishes to improve the reproducibility of the work that we publish. This form provides structure for consistency and transparency in reporting. For further information on Nature Portfolio policies, see our Editorial Policies and the Editorial Policy Checklist.

Statistics

For all statistical analyses, confirm that the following items are present in the figure legend, table legend, main text, or Methods section.

n/a	Confirmed	
☐	☒	The exact sample size (n) for each experimental group/condition, given as a discrete number and unit of measurement
☒	☐	A statement on whether measurements were taken from distinct samples or whether the same sample was measured repeatedly
☐	☒	The statistical test(s) used AND whether they are one- or two-sided<br>*Only common tests should be described solely by name; describe more complex techniques in the Methods section.*
☐	☒	A description of all covariates tested
☐	☒	A description of any assumptions or corrections, such as tests of normality and adjustment for multiple comparisons
☐	☒	A full description of the statistical parameters including central tendency (e.g. means) or other basic estimates (e.g. regression coefficient) AND variation (e.g. standard deviation) or associated estimates of uncertainty (e.g. confidence intervals)
☐	☒	For null hypothesis testing, the test statistic (e.g. F, t, r) with confidence intervals, effect sizes, degrees of freedom and P value noted<br>*Give P values as exact values whenever suitable.*
☐	☒	For Bayesian analysis, information on the choice of priors and Markov chain Monte Carlo settings
☒	☐	For hierarchical and complex designs, identification of the appropriate level for tests and full reporting of outcomes
☐	☒	Estimates of effect sizes (e.g. Cohen's d, Pearson's r), indicating how they were calculated

Our web collection on statistics for biologists contains articles on many of the points above.

Software and code

Policy information about availability of computer code

Data collection	We acquired the fMRI data using a 3T Philips Achieva MRI scanner. Visual stimuli were generated from client computers using Presentation software (Neurobehavioral Systems) controlled by a common server running the master script in MATLAB. The behavioural data were acquired through MATLAB R2020B.
Data analysis	Pre-processing and analysis of the fMRI data was performed using the FMRIB's Software Library (Functional MRI of the Brain, Oxford, UK). Analysis and modelling of behavioural data was performed through MATLAB code developed in the lab.

For manuscripts utilizing custom algorithms or software that are central to the research but not yet described in published literature, software must be made available to editors and reviewers. We strongly encourage code deposition in a community repository (e.g. GitHub). See the Nature Portfolio guidelines for submitting code & software for further information.

Data

Policy information about availability of data

All manuscripts must include a data availability statement. This statement should provide the following information, where applicable:

- Accession codes, unique identifiers, or web links for publicly available datasets
- A description of any restrictions on data availability
- For clinical datasets or third party data, please ensure that the statement adheres to our policy

The data that support the findings of this study is available on the Open Science Framework at this link: https://osf.io/sydea/

Human research participants

Policy information about studies involving human research participants and Sex and Gender in Research.

Reporting on sex and gender	Twenty-seven same sex pairs of adult human subjects participated in the experiment. Two couples were excluded for issues during the fMRI scan. The remaining couples were made of 7 males couples and 18 females couples.
Population characteristics	All subjects were right handed, had normal or corrected-to-normal vision and reported no history of psychiatric, neurological or major medical problems, and were free of psychoactive medications at the time of the study. The age range was 20:36.
Recruitment	All were recruited from the participants' database of the department of Psychology at the University of Glasgow. We excluded people outside the age range 18:40 as we were not interested in the effect of age.
Ethics oversight	Written informed consent was obtained in accordance with the Institute of Neuroscience and Psychology Ethics Committee at the University of Glasgow.

Note that full information on the approval of the study protocol must also be provided in the manuscript.

Field-specific reporting

Please select the one below that is the best fit for your research. If you are not sure, read the appropriate sections before making your selection.

☐ Life sciences ☒ Behavioural & social sciences ☐ Ecological, evolutionary & environmental sciences

For a reference copy of the document with all sections, see nature.com/documents/nr-reporting-summary-flat.pdf

Behavioural & social sciences study design

All studies must disclose on these points even when the disclosure is negative.

Study description	Couple of participants took part in a social game while quantitative behavioural and fMRI data were collected.
Research sample	Twenty-seven same sex pairs of adult human subjects participated in the experiment. Two couples were excluded for issues during the fMRI scan. The remaining couples were made of 7 males couples and 18 females couples. The age range was 20:36. The advert was directed at participants aged 18:40. The sample was representative of healthy young adults.
Sampling strategy	The number of couples to be scanned was determined based on a priori estimates of sample size necessary to ensure replicability on a task of similar length (110).
Data collection	In the fMRI sessions, participants were matched with an unfamiliar co-player they had not played with in the behavioural session and it was emphasised not to assume anything about their behaviour in the game. We did not use deception: participants briefly met before the experiment when a coin toss determined who would go into the scanner and who would play the game in a room adjacent to the fMRI control room where there was a computer running a MATLAB client for the game. A researcher familiar with the study was present outside the room. Visual stimuli were generated from client computers using Presentation software (Neurobehavioral Systems) controlled by a common server running the master script in MATLAB. The stimuli were presented to the players simultaneously. Each experiment was preceded by a short tutorial where players could experience a few trials in each of the three sessions to allow probing the effect of the variability in the task parameter. We acquired the fMRI data using a 3T Philips Achieva MRI scanner (Philips, Netherlands).
Timing	16/3/2017-15/12/2017
Data exclusions	One couple was removed for excessive head movements of the subject inside the scanner. Another couple was removed as the subject asked to interrupt the scan. One couple played only 30 trials (instead of 60) in the competitive condition as the scan was interrupted for a technical glitch.

Non-participation	No participants declined participation.
Randomization	Participants were matched in the game based on sex and availability for the different fMRI sessions. No other consideration was taken as we were not interested in the effect of age or other personal charateristic

Reporting for specific materials, systems and methods

We require information from authors about some types of materials, experimental systems and methods used in many studies. Here, indicate whether each material, system or method listed is relevant to your study. If you are not sure if a list item applies to your research, read the appropriate section before selecting a response.

Materials & experimental systems

n/a	Involved in the study
☒	☐ Antibodies
☒	☐ Eukaryotic cell lines
☒	☐ Palaeontology and archaeology
☒	☐ Animals and other organisms
☒	☐ Clinical data
☒	☐ Dual use research of concern

Methods

n/a	Involved in the study
☐	☐ ChIP-seq
☒	☐ Flow cytometry
☐	☒ MRI-based neuroimaging

Antibodies

Antibodies used	*Describe all antibodies used in the study; as applicable, provide supplier name, catalog number, clone name, and lot number.*
Validation	*Describe the validation of each primary antibody for the species and application, noting any validation statements on the manufacturer's website, relevant citations, antibody profiles in online databases, or data provided in the manuscript.*

Eukaryotic cell lines

Policy information about cell lines and Sex and Gender in Research

Cell line source(s)	*State the source of each cell line used and the sex of all primary cell lines and cells derived from human participants or vertebrate models.*
Authentication	*Describe the authentication procedures for each cell line used OR declare that none of the cell lines used were authenticated.*
Mycoplasma contamination	*Confirm that all cell lines tested negative for mycoplasma contamination OR describe the results of the testing for mycoplasma contamination OR declare that the cell lines were not tested for mycoplasma contamination.*
Commonly misidentified lines (See ICLAC register)	*Name any commonly misidentified cell lines used in the study and provide a rationale for their use.*

Palaeontology and Archaeology

Specimen provenance	*Provide provenance information for specimens and describe permits that were obtained for the work (including the name of the issuing authority, the date of issue, and any identifying information). Permits should encompass collection and, where applicable, export.*
Specimen deposition	*Indicate where the specimens have been deposited to permit free access by other researchers.*
Dating methods	*If new dates are provided, describe how they were obtained (e.g. collection, storage, sample pretreatment and measurement), where they were obtained (i.e. lab name), the calibration program and the protocol for quality assurance OR state that no new dates are provided.*

☐ Tick this box to confirm that the raw and calibrated dates are available in the paper or in Supplementary Information.

Ethics oversight	*Identify the organization(s) that approved or provided guidance on the study protocol, OR state that no ethical approval or guidance was required and explain why not.*

Note that full information on the approval of the study protocol must also be provided in the manuscript.

Animals and other research organisms

Policy information about studies involving animals; ARRIVE guidelines recommended for reporting animal research, and Sex and Gender in Research

Laboratory animals	*For laboratory animals, report species, strain and age OR state that the study did not involve laboratory animals.*
Wild animals	*Provide details on animals observed in or captured in the field; report species and age where possible. Describe how animals were caught and transported and what happened to captive animals after the study (if killed, explain why and describe method; if released, say where and when) OR state that the study did not involve wild animals.*
Reporting on sex	*Indicate if findings apply to only one sex; describe whether sex was considered in study design, methods used for assigning sex. Provide data disaggregated for sex where this information has been collected in the source data as appropriate; provide overall numbers in this Reporting Summary. Please state if this information has not been collected. Report sex-based analyses where performed, justify reasons for lack of sex-based analysis.*
Field-collected samples	*For laboratory work with field-collected samples, describe all relevant parameters such as housing, maintenance, temperature, photoperiod and end-of-experiment protocol OR state that the study did not involve samples collected from the field.*
Ethics oversight	*Identify the organization(s) that approved or provided guidance on the study protocol, OR state that no ethical approval or guidance was required and explain why not.*

Note that full information on the approval of the study protocol must also be provided in the manuscript.

Clinical data

Policy information about clinical studies

All manuscripts should comply with the ICMJE guidelines for publication of clinical research and a completed CONSORT checklist must be included with all submissions.

Clinical trial registration	*Provide the trial registration number from ClinicalTrials.gov or an equivalent agency.*
Study protocol	*Note where the full trial protocol can be accessed OR if not available, explain why.*
Data collection	*Describe the settings and locales of data collection, noting the time periods of recruitment and data collection.*
Outcomes	*Describe how you pre-defined primary and secondary outcome measures and how you assessed these measures.*

Dual use research of concern

Policy information about dual use research of concern

Hazards

Could the accidental, deliberate or reckless misuse of agents or technologies generated in the work, or the application of information presented in the manuscript, pose a threat to:

No	Yes	
☐	☐	Public health
☐	☐	National security
☐	☐	Crops and/or livestock
☐	☐	Ecosystems
☐	☐	Any other significant area

Experiments of concern

Does the work involve any of these experiments of concern:

No	Yes	
☐	☐	Demonstrate how to render a vaccine ineffective
☐	☐	Confer resistance to therapeutically useful antibiotics or antiviral agents
☐	☐	Enhance the virulence of a pathogen or render a nonpathogen virulent
☐	☐	Increase transmissibility of a pathogen
☐	☐	Alter the host range of a pathogen
☐	☐	Enable evasion of diagnostic/detection modalities
☐	☐	Enable the weaponization of a biological agent or toxin
☐	☐	Any other potentially harmful combination of experiments and agents

ChIP-seq

Data deposition

☐ Confirm that both raw and final processed data have been deposited in a public database such as GEO.

☐ Confirm that you have deposited or provided access to graph files (e.g. BED files) for the called peaks.

Data access links<br>*May remain private before publication.*	*For "Initial submission" or "Revised version" documents, provide reviewer access links. For your "Final submission" document, provide a link to the deposited data.*
Files in database submission	*Provide a list of all files available in the database submission.*
Genome browser session<br>(e.g. UCSC)	*Provide a link to an anonymized genome browser session for "Initial submission" and "Revised version" documents only, to enable peer review. Write "no longer applicable" for "Final submission" documents.*

Methodology

Replicates	*Describe the experimental replicates, specifying number, type and replicate agreement.*
Sequencing depth	*Describe the sequencing depth for each experiment, providing the total number of reads, uniquely mapped reads, length of reads and whether they were paired- or single-end.*
Antibodies	*Describe the antibodies used for the ChIP-seq experiments; as applicable, provide supplier name, catalog number, clone name, and lot number.*
Peak calling parameters	*Specify the command line program and parameters used for read mapping and peak calling, including the ChIP, control and index files used.*
Data quality	*Describe the methods used to ensure data quality in full detail, including how many peaks are at FDR 5% and above 5-fold enrichment.*
Software	*Describe the software used to collect and analyze the ChIP-seq data. For custom code that has been deposited into a community repository, provide accession details.*

Flow Cytometry

Plots

Confirm that:

☐ The axis labels state the marker and fluorochrome used (e.g. CD4-FITC).

☐ The axis scales are clearly visible. Include numbers along axes only for bottom left plot of group (a 'group' is an analysis of identical markers).

☐ All plots are contour plots with outliers or pseudocolor plots.

☐ A numerical value for number of cells or percentage (with statistics) is provided.

Methodology

Sample preparation	*Describe the sample preparation, detailing the biological source of the cells and any tissue processing steps used.*
Instrument	*Identify the instrument used for data collection, specifying make and model number.*

Software

Describe the software used to collect and analyze the flow cytometry data. For custom code that has been deposited into a community repository, provide accession details.

Cell population abundance

Describe the abundance of the relevant cell populations within post-sort fractions, providing details on the purity of the samples and how it was determined.

Gating strategy

Describe the gating strategy used for all relevant experiments, specifying the preliminary FSC/SSC gates of the starting cell population, indicating where boundaries between "positive" and "negative" staining cell populations are defined.

☐ Tick this box to confirm that a figure exemplifying the gating strategy is provided in the Supplementary Information.

Magnetic resonance imaging

Experimental design

Design type

Task and event related.

Design specifications

we run 3 blocks of 60 trials each, each trial last about 10 seconds and there were small gaps (~30 seconds) between blocks.

Behavioral performance measures

We recorded button presses indicating choices in the game. Participants responded in virtually all trials (response rate >99%)

Acquisition

Imaging type(s)

functional MRI

Field strength

3T

Sequence & imaging parameters

we collected functional Echo-Planar-Imaging (EPI) data using an 32-channel SENSE head coil with an anterior–posterior fold over direction (SENSE factor: 2.3; repetition time: 1.5 s; echo time: 40 ms; number of slices: 40; number of voxels: 68 × 68; in-plane resolution: 3 × 3 mm; slice thickness: 3 mm; flip angle: 80°)

Area of acquisition

whole brain

Diffusion MRI ☐ Used ☐ Not used

Preprocessing

Preprocessing software

Pre-processing of our data was performed using the FMRIB's Software Library (Functional MRI of the Brain, Oxford, UK) and included: head-related motion correction, slice-timing correction, high-pass filtering (>100 s), and spatial smoothing (with a Gaussian kernel of 8 mm full-width at half maximum).

Normalization

To register our EPI image to standard space, we first transformed the EPI images into each individual's high-resolution space with a linear six-parameter rigid body transformation. We then registered the image to standard space (Montreal Neurological Institute, MNI) using FMRIB's Non-linear Image Registration Tool with a resolution warp of 10 mm.

Normalization template

MNI152_T1_2mm_brain

Noise and artifact removal

preprocessing included head-related motion correction, slice-timing correction, high-pass filtering (>100 s), and spatial smoothing (with a Gaussian kernel of 8 mm full-width at half maximum). We also acquired a B0 map using a multi-shot gradient echo sequence which was subsequently used to correct for distortions in the EPI data due to B0 inhomogeneities (echo time: 2.3 ms; delta echo time: 5 ms; isotropic resolution: 3 mm; matrix: 68 × 68 × 32; repetition time: 383 ms; flip angle: 90°).

Volume censoring

we didn't censor volumes

Statistical modeling & inference

Model type and settings

We performed whole-brain statistical analyses of functional data using a multilevel approach within the generalized linear model (GLM) framework, as implemented in FSL through the FEAT module:
Y=Xβ+ ε=?β_1 X?_1+?β_2 X?_2+?...+β_N X?_N+ ε
where Y is a T×1 (T time samples) column vector containing the times series data for a given voxel, and X is a T × N (N regressors) design matrix with columns representing each of the psychological regressors convolved with a hemodynamic response function specific for human brains (113, 114). β is a N × 1 column vector of regression coefficients and ε a T × 1 column vector of residual error terms. Using this framework we initially performed a first-level fixed effects analysis to process each individual experimental run which were then combined in a second-level mixed-effects analysis (FLAME 1 + 2) treating session as a random effects (we also had a similar number of sessions across subjects). For all analysis, we performed a cluster inference using a cluster-defining threshold of |Z| > 3.1 with a FWE-corrected threshold of P = 0.001. Time series statistical analysis was carried out using FMRIB's improved linear model with local autocorrelation correction.

Effect(s) tested

Our main GLM model included four unmodulated stick regressors aligned with (i) the beginning of the trial (TRIAL in suppl.

Effect(s) tested	Table 2) (ii) the player response (PR) (iii) the time at which the response of the co-player was revealed (OR) (iv) the time at which the target appeared (TARGET). Additionally, we included six regressors capturing trial-by-trial specific information: (1) a stick function at (i) the beginning of the trial parametrically modulated by the expected position of the co-player as derived through the average of the prior distribution for that trial obtained from the Bayesian model (PriorPos). (2) a stick function at (ii) response time modulated by trial by trial changes in the level of cooperation chosen by the player (Pcoop). (3 and 4) two stick functions at (iii) the time at which the response of the co-player was revealed parametrically modulated respectively by the value of the KL divergence between prior and posterior computed in that trial (absPE) and its sign (signPE). The latter could only take the value +1 and -1. Finally (5 and 6)) two stick functions at (iv) the time at which the target appeared parametrically modulated respectively by the value of the reward allocated in the trial (Rew) and one signalling whether the player won or lose (Win). The latter could only take the value +1 and -1. All parametrically modulated regressors were z-scored.

Specify type of analysis: ☐ Whole brain ☐ ROI-based ☒ Both

Anatomical location(s)	We selected region of interest in regions significantly activated in response to the regressors of interest (signPE absPE and pcoop). To quantify the modulation of the activity across conditions, we extracted the average signal of the neural activation for all three social contexts in regions of interest (ROIs), defined as either three or five-voxel radius spherical masks placed centred on the peak of the activations at the group level.
Statistic type for inference (See Eklund et al. 2016)	For all analysis, we performed a cluster inference using a cluster-defining threshold of $\|Z\| > 3.1$ with a FWE-corrected threshold of $P = 0.001$.
Correction	FWE-corrected threshold of $P = 0.001$

Models & analysis

n/a	Involved in the study
☒	☐ Functional and/or effective connectivity
☒	☐ Graph analysis
☒	☐ Multivariate modeling or predictive analysis
